# Dronabinol has preferential antileukemic activity in acute lymphoblastic and myeloid leukemia with lymphoid differentiation patterns

**DOI:** 10.1186/s12885-015-2029-8

**Published:** 2016-01-16

**Authors:** Kerstin Maria Kampa-Schittenhelm, Olaf Salitzky, Figen Akmut, Barbara Illing, Lothar Kanz, Helmut Rainer Salih, Marcus Matthias Schittenhelm

**Affiliations:** University Hospital Tübingen, Dept. of Oncology, Hematology, Rheumatology, Immunology and Pulmology, Tübingen, Germany

**Keywords:** Delta9-Tetrahydrocannabinol, Dronabinol, THC, Leukemia, AML, ALL

## Abstract

**Background:**

It has been previously demonstrated in several cancer models, that Dronabinol (THC) may have anti-tumor activity – however, controversial data exists for acute leukemia. We have anecdotal evidence that THC may have contributed to disease control in a patient with acute undifferentiated leukemia.

**Methods:**

To test this hypothesis, we evaluated the antileukemic efficacy of THC in several leukemia cell lines and native leukemia blasts cultured ex vivo. Expression analysis for the CB1/2 receptors was performed by Western immunoblotting and flow cytometry. CB-receptor antagonists as well as a CRISPR double nickase knockdown approach were used to evaluate for receptor specificity of the observed proapoptotic effects.

**Results:**

Meaningful antiproliferative as well as proapoptotic effects were demonstrated in a subset of cases – with a preference of leukemia cells from the lymphatic lineage or acute myeloid leukemia cells expressing lymphatic markers. Induction of apoptosis was mediated via CB1 as well as CB2, and expression of CB receptors was a prerequisite for therapy response in our models. Importantly, we demonstrate that antileukemic concentrations are achievable in vivo.

**Conclusion:**

Our study provides rigorous data to support clinical evaluation of THC as a low-toxic therapy option in a well defined subset of acute leukemia patients.

**Electronic supplementary material:**

The online version of this article (doi:10.1186/s12885-015-2029-8) contains supplementary material, which is available to authorized users.

## Background

Delta9-Tetrahydrocannabinol is the major psychoactive constituent of Cannabis sativa and signals through G-protein-coupled cannabinoid receptors (CB).

The CB1 receptor is predominantly abundant in brain tissues [1]. In contrast, the CB2 receptor was initially described in the lymphatic system [2], but is also expressed in other tissues such as brain [3], brain endothelium [4], bone [5] or skin [6].

While the central CB1 receptor accounts for the psychotropic, analgetic, and orectic effects, the dominantly peripheral CB2 receptor is linked to immunomodulation [7] and regulation of bone mass [5] among other functions.

Despite the broadly acknowledged potential of cannabinoid agonists with regard to effective relief of tumor or neuropathic pain, muscular spasm or nausea—combined with an excellent safety profile and moderate side-effects—clinical use is very restricted in most countries due to the unwanted psychoactive effects (reviewed by Pertwee [8]). The natural (−)-Δ^9^-Tetrahydrocannabinol isomer dronabinol (further referred to as THC) is a potent pan-cannabinoid receptor (CB1/2) agonist, which gained FDA-approval in the United States as Marinol® for the treatment of chemotherapy-induced nausea and vomiting or stimulation of appetite in AIDS patients.

Moreover and importantly, there is evidence for growth-inhibiting effects in tumor models, including animal models, arguing for the use of cannabinoids as low-toxic anticancer therapeutics (reviewed by Guzman [9]).

Anecdotal evidence has lead us to speculate that THC may have contributed to disease control in a patient with acute undifferentiated leukemia. Indeed, previous reports suggest a proapoptotic antitumor effect of CB-agonists on acute leukemia cells in vitro [10–12]. These studies concentrate on the analysis of the Jurkat T-lymphoblastic cell line (which was established from the peripheral blood of a patient suffering from acute T-lymphoblastic leukemia in 1976; see also http//:https://www.dsmz.de). However, the mechanism of action is controversially discussed in these studies (CB1 versus CB2 mediation) [11, 12].

Even more controversially, other studies suggest a hematopoietic growth advantage mediated via CB2 activation – and utmost challenging, characterize CB2 as an oncoprotein linked to (myeloid) leukemogenesis [13–15].

We now provide data demonstrating potent antileukemic efficacy of THC in acute leukemia cell lines in vitro as well as freshly harvested native leukemia blasts cultured ex vivo. Notably, antiproliferative as well as proapoptotic effects are preferentially seen in leukemia cells of the lymphatic lineage or in acute myeloid leukemia cells expressing lymphatic markers.

## Results

### THC inhibits cellular proliferation in lymphatic and myeloid leukemia cell lines

**Fig. 1:**
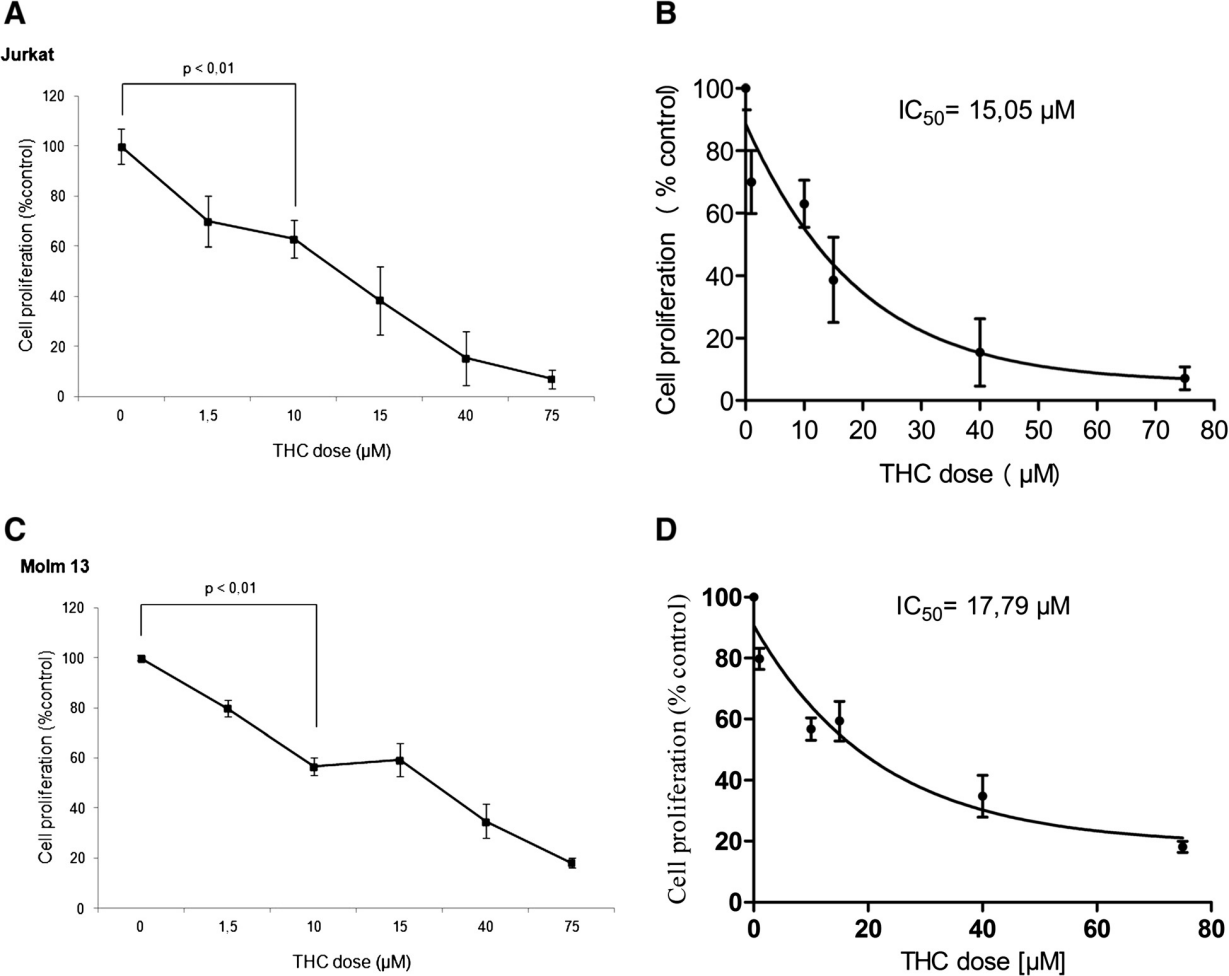
Photometric XTT-analysis assaying metabolic active cells in dependence of THC concentration. Representative dose-effect curves for Jurkat (**a**) and MOLM13 (**c**) cells treated with THC in a dose-dependent manner are shown on. Student’s *t*-test analysis reveals significant reduction of proliferating cells as indicated for two doses (statistical significance at *p* < 0.05). Experiments were performed in triplicates. Linear regression analysis was performed to compute IC_50s_ for both cell lines (**b** and **d**)

In analogy to previous reports, we used the T-lymphoblastic leukemia cell line Jurkat to reconfirm whether THC is capable to inhibit cellular proliferation in an acute leukemia cell model. THC was administered for 72 h in a dose dependent manner and the antiproliferative effect, measured as the reduction of XTT metabolism in correlation to an untreated negative control, was measured accordingly. THC produced significant and dose-dependent inhibition of cellular proliferation (Fig. 1a) with a computed IC_50_ ~ 15 μM in a non-linear regression analysis (Fig. 1b).

To determine, whether the observed effects are unique to the Jurkat cell line, we also tested an acute myeloid leukemia cell line, MOLM13 – and found similar antiproliferative effects with an IC_50_ ~ 18 μM (Fig. 1c, d).

At the higher tested doses >50 μM, virtual no metabolic activity was observed for both Jurkat as well as MOLM13 cells – arguing that cells are not viable and may have been directed to programmed cell death. In this context, it has been previously described, that cannabinoid agonists are capable to induce apoptosis in tumor cells [16].

### THC induces apoptosis in leukemia cell lines

We addressed this question in an annexin V-based flow cytometry assay and treated Jurkat as well as MOLM13 cells with increasing concentrations of THC for 48 h.

**Fig. 2:**
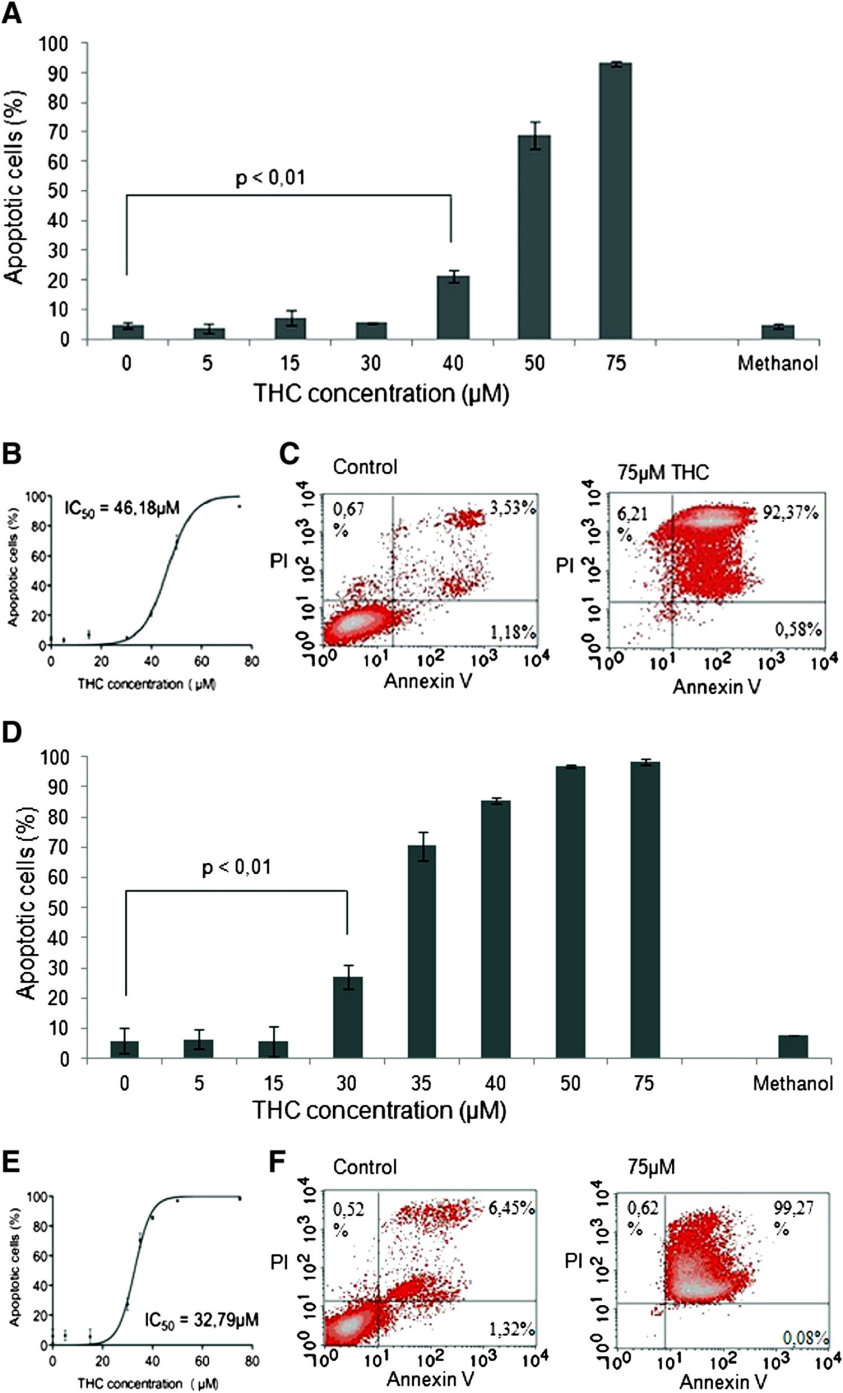
Flow cytometric apoptosis assay measuring early apoptotic (annexin V) and later phase apoptotic cells (propidium iodide) after exposure of Jurkat (**a**-**c**) or MOLM13 (**d**-**f**) cells to THC. Dose-effect curves for Jurkat (**a**) and MOLM13 (**d**) cells treated with THC in a dose-dependent manner are shown. Student’s *t*-test demonstrates significance (*p* < 0.05) of induction of apoptosis at 46 μM (Jurkat), resp. 32 μM (MOLM13). Experiments were performed in triplicates. Non-linear regression analysis was performed to compute IC_50s_ (**b**, **e**). Flow cytometry raw data are shown for Jurkat (**c**) and MOLM13 (**f**) cells demonstrating overwhelming induction of apoptosis in the highest tested doses – with no effect for methanol as drug carrier at the highest tested dose

For Jurkats, we were able to demonstrate dose-dependent induction of apoptosis with significant p values starting at 40 μM in a Student’s *t*-test (Fig. 2a). IC_50_ was computed in a non-linear regression analysis at ~46 μΜ (Fig. 2b). No signs of cell cycle arrest with abrogation of the proapoptotic effect in higher doses [17] were seen: At the highest tested dose, 75 μM, a virtual complete kill of the entire population was observed (Fig. 2c).

Additional annexin V-staining data is provided in Additional file 1: Figure S1, demonstrating dose-dependent induction of early apoptosis in Jurkat cells treated with THC for 10 h.

It has been previously demonstrated for Jurkat leukemia cells, that THC-mediated induction of apoptosis is linked to the intrinsic, mitochondrial pathway [10]. We confirmed this finding in Western immunoblots showing cleavage of caspases 3 and 9 upon treatment with THC (Fig. 5c and Additional file 2: Figure S2). Cleaved caspase 9 is known as a central mediator of the intrinsic mitochondrial apoptosis pathway.

Similarly, MOLM13 cells underwent induction of apoptosis in response to THC with a computed IC_50_ ~ 38 μM and complete kill of cells at 75 μM (Fig. 2d–f).

A drug-carrier (i.e. methanol) control assay did not reveal any significant proapoptotic effects at the highest concentration used with the THC-dilution experiments.

To diminish individual cell line-specific effects, we expanded our analysis to other leukemia cell line models. For our experiments, we used MOLM14 cells, a sister cell line of MOLM13 derived from the same patient, as well as independent acute myeloid leukemia cell lines (MV4-11, M0-7e, HL60), the core binding factor cell line Kasumi1 and the acute blast crisis CML cell line K562.

All cell lines were treated with THC in a dose dependent manner and induction of apoptosis was measured after 24 and 48 h.

Together, THC was capable of inducing apoptosis in all leukemia cell lines—whereas IC_50s_ differed in between the tested cell lines. A summary of computed IC_50s_ is provided with Table 1. Dose-effect plots and dose-regression analysis for each cell line are provided as supplemental data (Additional file 3: Figure S3, Additional file 4: Figure S4, Additional file 5: Figure S5, Additional file 6: Figure S6, Additional file 7: Figure S7, Additional file 8: Figure S8).

**Table 1:** Sensitivity of leukemia cell lines in response to THC

Patient No.	Phenotype			THC response	
*Entitiy*	*lineage dependency is marked (+) aberrantly expressed antigens are separately indicated*			*% viable cells at 50 μM*	*IC50 (μM)*
	T-lymphatic	B-lymphatic	myeloid		
K562	−	−	+	87	62
M07e	−	−	+	81	59
HL60	CD4	−	+	15	38
Kasumi1	CD4	−	+	14	35
MV4-11	CD4	−	+	3	39
MOLM14	CD4	−	+	18	44
MOLM13	CD4	−	+	3	33
Jurkat	+	−	−	31	46

### THC reduces the proportion of viable cells cultured ex vivo

We next tested native leukemia blasts cultured ex vivo, with regard to the antileukemic sensitivity after exposure to THC with doses in the range of IC_50s_ for the Jurkat cell line.

**Fig. 3:**
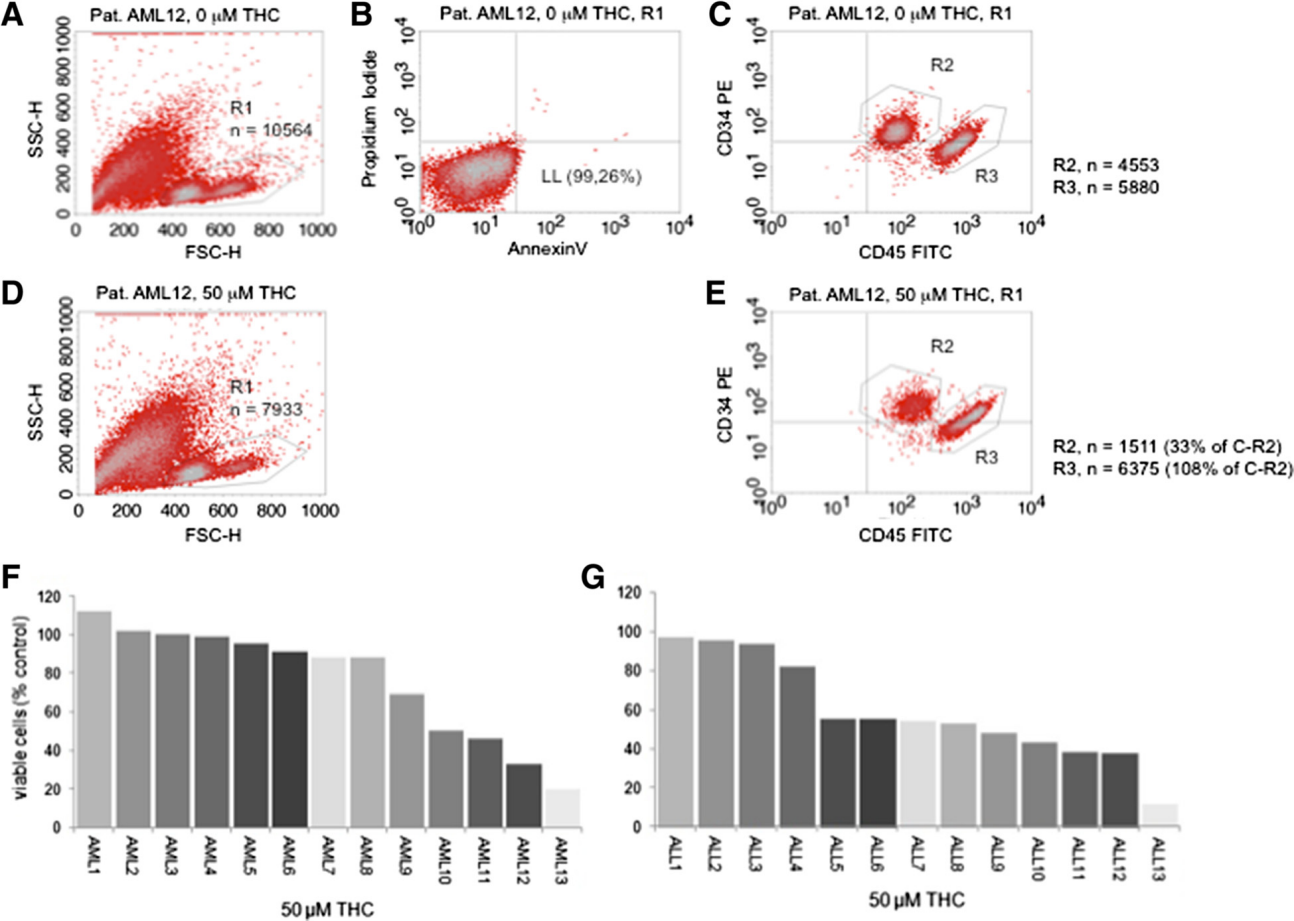
Reduction of the viable leukemia cohort upon treatment with THC. **a** FSC/SSC scatter plot was used to gate (R1) the viable cell population. Counted cells (total) *n* = 30,000. **b** Viability of the R1-gated population was confirmed in an annexin V/PI-based apoptosis assay (viable population located in the lower left (LL) section of a quadrant plot). **c** Immunophenotyping assay to distinguish the CD34 (PE conjugated) and/or CD45low (FITC conjugated) positive leukemia population is shown. This cohort was followed prior to and 48 h after exposure to THC to determine reduction of viable cells in response to THC (**d** and **e**). A representative patient sample is shown. Percental waterfall plots are provided for AML (**f**) and ALL (**g**) for all tested patient samples

As a relatively high basal proportion of dead/apoptotic cells was present in the freshly harvested and cultured cells, which is a commonly observed problem in ex vivo cell cultures, we used a flow cytometry-based assay as recently established by our group (Kampa-Schittenhelm et al. [18]) measuring reduction of the viable cell proportion in a FSC/SSC scatter plot. To ensure that the gated population is viable an annexin V/PI-based assay was performed simultaneously using THC-naïve cells. The viable cell fraction was defined as absence of annexin V or PI positivity and the gate was set accordingly. Further, immunophenotyping was set up to confirm the leukemic character of the gated population (i.e. CD45low+/-CD34 positivity). Reduction of the viable cell fraction was measured 48 h after THC exposure compared to treatment-naive parental cells. Density dot plots of a representative patient sample are provided in Fig. 3a–e.

Dose-effect waterfall bar graphs demonstrating reduction of viable cells in lymphatic as well as myeloid leukemia patient samples are provided with Fig. 3f (myeloid leukemia) and Fig. 3g (lymphatic leukemia). In general, leukemias with lymphatic differentiation were more sensitive to THC—with 9/13 (69 %) patients showing an at least ~50 % reduction of viable cells at 50 μM. In contrast, only 4/13 (31 %) patients with AML demonstrated a ≥50 % reduction of the viable cell proportion.

### Response to THC correlates with expression of CB1 and CB2 receptors

**Fig. 4:**
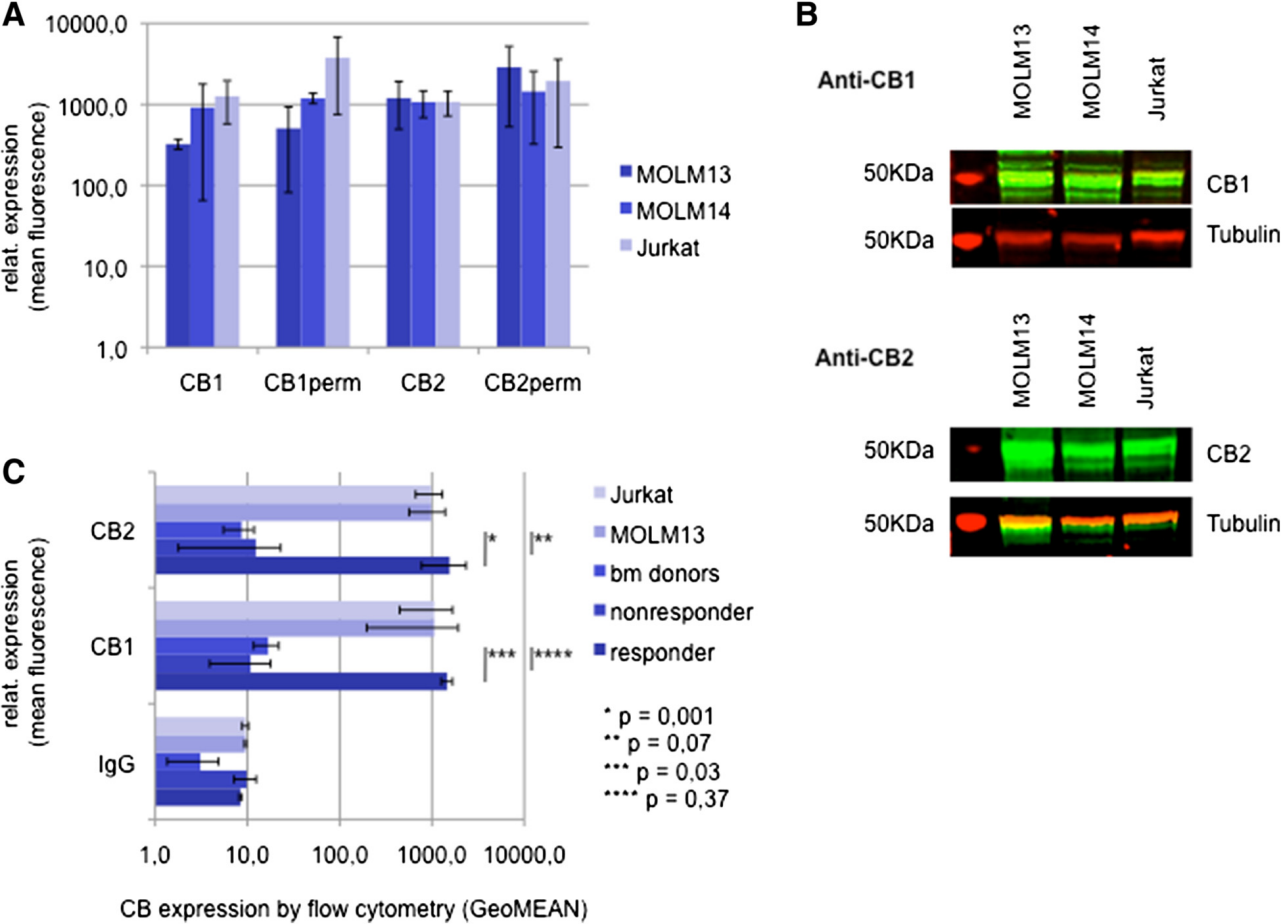
Expression of CB1 and CB2 in acute leukemia. **a** FACS flow cytometry based analysis of intracellular (CB1/2 perm) and extracellular CB expression levels in MOLM and Jurkat cell lines. **b** Western immunoblotting expression analysis of CB1 and CB2 in Jurkat and MOLM leukemia cell lines. The major isoform of CB1 (1a long) has a molecular weight of 52 KDa. CB2 is expected at 40-50 KDa. **c** Exracellular CB1/CB2 expression levels of native leukemia cells (*n* = 12) and comparatively bone marrow donors (*n* = 10) and the Jurkat and MOM13 cell lines as assessed by flow cytometry. The responder/nonresponder cohort (*n* = 4, resp. *n* = 8) contains patient samples responsive/non-responsive towards THC ex vivo. (*-****) statistical significance at *p* < 0.05 (Student’s *t*-test)

To evaluate, whether response to THC correlates with cannabinoid receptor expression, we measured protein expression levels of CB1 and CB2 on all available patient samples using a flow cytometry-based assay. Antibody-specificity was validated by Western immunoblots and flow cytometry analysis using MOLM and Jurkat cell lines (Fig. 4a–b).

Marked CB1 as well as CB2 expression was confirmed in 4/12 evaluated patients. Interestingly, expression of CB1 as well as CB2 was individually but equally elevated in these patients. The remaining 8 patients showed significantly lower expression levels of either of the receptors (Fig. 4c). Comparative evaluation of CB receptor expression levels in 10 healthy bone marrow donors revealed similar low expression levels.

Notably, correlation of CB-expression levels with responders to THC (defined as an apoptosis rate of at least 20 % upon treatment with THC for 48 h) revealed that expression of the cannabinoid receptors is a definite prerequisite to achieve any proapoptotic effect in native leukemia blasts.

### The proapoptotic effect of THC is mediated via CB1 – as well as CB2

As both receptors were equally increased or diminished in all tested cell lines and patient samples, we asked whether the observed proapoptotic effect can be linked to one specific receptor.

**Fig. 5:**
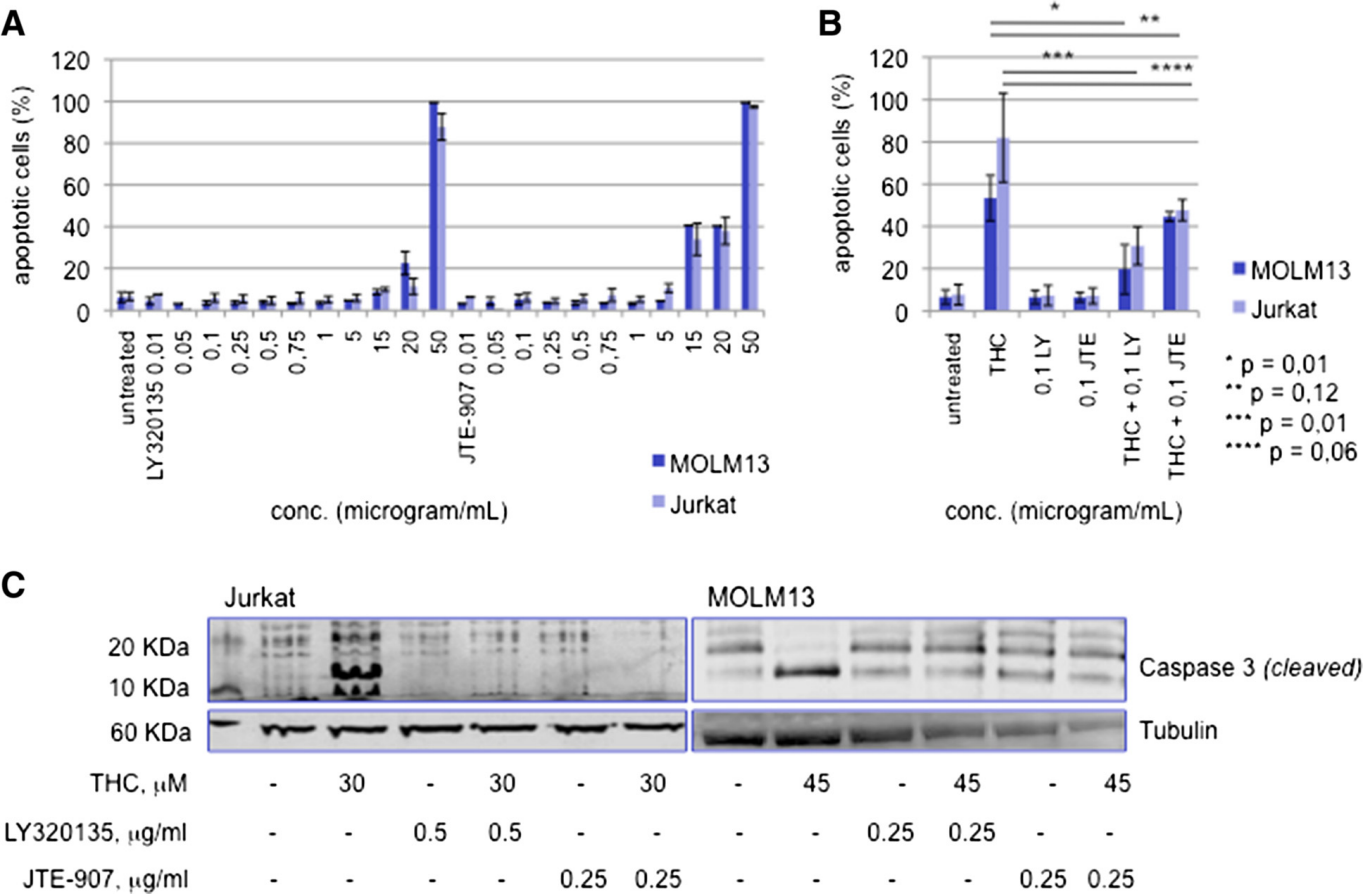
Proapoptotic effect of THC is mediated via CB1 and CB2. **a** The CB1 antagonist LY320135 and a selective CB2 inverse ligand agonist (JTE-907) were tested in dose-dependent dilution series. Dose-effect plots from an apoptosis annexin V-based flow cytometry assay are shown. **b** MOLM13 and Jurkat cells were pretreated with either antagonist (LY, LY320135; JTE, JTE-907) for 12 h and THC was administered for another 48 h (30 μM for Jurkat and 45 μM for MOLM13 cells). Induction of apoptosis was analyzed as described above. (*-****) statistical significance at *p* < 0.05 (Student’s *t*-test). **c** Western immunoblotting of cleaved caspase 3 in response to THC +/- preexposition to LY320135 or JTE-907 is shown.

We established an assay to specifically block the CB1 or CB2 receptor prior to exposure of leukemia cells to THC and used MOLM13 or Jurkat cells as a myeloid, respective lymphoid leukemia model:

LY320135, a highly selective cannabinoid receptor antagonist with a 70-fold higher affinity to CB1 than CB2 and a selective CB2 inverse ligand agonist (JTE-907) were first tested in dose-dependent dilution series in both cell lines to determine the optimal concentration without an intrinsic cell toxic effect (Fig. 5a).

Jurkat or MOLM13 cells were next treated with subtoxic doses of either LY320135 or JTE-907 at 0,1 μg/ml for 12 h. THC was then administered at ~ IC_50_ doses and cells were incubated for an additional 48 h. Notably, both inhibitors were able to abrogate THC-mediated induction of apoptosis in Jurkat cells as well as the MOLM13 cell line (Fig. 5b). As statistical analysis closely failed significance for CB2-interfered cell strains, we set up an alternative approach to confirm CB1- as well as CB2-dependency of the proapoptotic effect in leukemia cells: A knockout transfection approach was established using a CRISPR double nickase plasmid selectively encoding for CB1 or CB2. Puromycin selection was used to create stable CB1, resp. CB2 knockout cell strains of the Jurkat leukemia cell line. Importantly, knockdown of CB1 as well as CB2 resulted in highly significant abrogation of proapoptotic effects upon treatment with THC (see Additional file 9: Figure S9), supporting the finding of a direct role of either of the cannabinoid receptors in induction of apoptosis in acute leukemia models.

To confirm rescue from induction of apoptosis on the protein level, cleavage of caspase 3 (as an indicator of activated apoptosis signal transduction pathways) was determined by western immunoblot experiments. Indeed, THC-treated MOLM13 as well as Jurkat cells were successfully rescued from caspase 3 cleavage after pre-treatment of cells with LY320135 or JTE-907 (Fig. 5c).

### Response to THC is higher in leukemia blasts expressing lymphatic markers

As demonstrated in Fig. 3, responses to THC were predominantly seen in acute leukemia entities derived from the lymphatic lineage. However, there was a subset of myeloid leukemia patient samples that had considerable sensitivity towards THC as well.

In an attempt, to further define the cohort responsive towards THC, we performed a systematic review of all available expression markers obtained at diagnosis—and found that most sensitive AML samples aberrantly expressed (T-) lymphoid differentiation markers (Table 2).

**Table 2:** Sensitivity of native leukemia blasts in response to THC

Patient No.	Phenotype			THC response
*Entity*	lineage dependency is marked (+) aberrantly expressed antigens are separately indicated			% viable cells at 50 μM
	T-lymphatic	B-lymphatic	myeloid	
AML-1				
*sAML (MDS)*	N/A	N/A	+	100
AML-2				
*AML, FLT3-ITD*	−	−	+	100
AML-3				
*AML NOS*	(CD3)	−	+	100
AML−4				
*APL*	−	−	+	99
AML-5				
*AML, FLT3-ITD*	−	−	+	96
AML-6				
*AML, FLT3-ITD*	−	CD19	+	91
AML-7				
*AML NOS (M0)*	−	−	+	89
AML-8				
*AML, FLT3-ITD*	−	−	+	88
AML-9				
*N/A*	N/A	N/A	+	69
AML-10	CD7			
*AML, FLT3-ITD*	CD56	−	+	50
*AML-11*	CD7			
*sAML (MDS)*	CD5	−	+	46
AML-12	(CD7)			
*AML NOS (M0)*	(CD5)	−	+	33
AML-13	CD7			
*CBF AML*	CD5	−	+	20
ALL-1				
*(c) B-ALL*	−	+	−	97
ALL-2			CD33	
*pre B-ALL*	−	+	CD13	96
ALL-3				
*(c) B-ALL*	−	+	CD33	94
ALL-4				
*pre-B-ALL*	−	+	−	82
ALL-5				
*(c) B-ALL*	−	+	−	55
ALL-6				
*Cortical T-ALL*	+	CD79	−	55
ALL-7				
*(c) B-ALL*	−	+	−	54
ALL-8		+	CD33	
*pre B-ALL*	−	CD10	CD13	53
ALL-9				
*(c) B-ALL*	−	+	−	48
ALL-10				
*(c) B-ALL*	−	+	−	43
ALL-11				
*Cortical T-ALL*	+	−	−	38
ALL-12			CD33	
*(c) B-ALL*	−	+	CD13	37
ALL-13	CD56			
*pre B-ALL*	CD1a	+	CD13	12

In this context, it is utmost remarkable, that all analyzed leukemia cell lines with higher sensitivity towards THC aberrantly express T-lymphatic antigens as well (summarized in Table 1, see DSMZ homepage and Matsuo et al. [19] for expression profiles of cell lines).

However, as ALL samples with sensitivity towards THC were not restricted to the T-lineage, the observation of linking T-cell markers with THC-response may be biased due to the limited number of samples analyzed – and AML cohorts expressing B-cell markers may respond to THC as well. In our tested cohort, the only case expressing a B-differentiation marker (CD19, AML-6) did not show significant sensitivity towards THC up to 50 μM.

## Discussion

Treatment outcome for acute leukemia in adults is still unsatisfactory for most entities. Besides disease-specific limitations such as high-risk genomic or chromosomal aberrations, comorbidities need to be addressed, especially in the increasing elderly population, restricting therapeutic options to epigenetic approaches, symptomatic cytoreduction or best supportive care.

We here reveal a novel aspect of dronabinol, a cannabinoid derivative, which displays remarkable antiproliferative as well as proapoptotic efficacy in a distinct leukemia patient cohort - in vitro and in ex vivo native leukemia blasts. It has been previously reported that cannabinoids display anticancer properties. However, due to legal issues the use and exploration of such agents is highly limited in many countries. Definition of dosing and entities benefitting from these agents remain vague and despite mounting evidence regarding their anti-tumorous effects cannabinoids have not been further developed as anticancer agents.

Even more challenging, controversial data suggest that cannabinoid agonists may foster tumorigenesis in some entities: For an acute myeloid leukemia model it has been demonstrated that CB2 has oncogene properties abrogating myeloid differentiation [13, 20].

We now provide rigorous proof-of-principle data demonstrating that (A) dronabinol has antiproliferative as well as proapoptotic efficacy in a broad spectrum of acute leukemia cell lines and native blasts cultured ex vivo and (B) this effect was preferentially observed in blasts with lymphoid differentiation or myeloid blasts aberrantly expressing lymphatic antigens. (C) The proapoptotic effect of dronabinol is mediated via CB1 as well as CB2 – and expression of the CB receptors is a prerequisite for therapy response. (D) Antitumor efficacy is dose-dependent and achievable in vivo.

Despite numerous reports on the anti-cancerous efficacy of THC the mechanisms of action as well as defined responder populations still remain unclear. Our data demonstrating antiproliferative as well as proapoptotic efficacy in defined acute leukemia models as well as ex vivo patient samples thereby aims to define a patient sample cohort potentially profiting from dronabinol therapy. The observation that lymphoid blasts or myeloid samples expressing lymphatic markers are more sensitive towards THC is extremely valuable for therapeutic decisions and the observed lineage-dependency might explain the controversial results observed for cannabinoid activation in acute leukemia models in the past. But studies on a larger patient cohort are necessary to verify our observation and future studies will have to address the underlying mechanisms.

The mediation via both cannabinoid receptors CB1 and CB2 was verified using two different strategies – by transient silencing receptor activity via specific antagonists and by CRISPR double nickase knockdown. Our in vitro data is thereby backed up by the observation that all patient samples sensitive towards THC presented with high protein expression levels of CB1 and CB2 receptors whereas vice versa all non-responder displayed only low CB1/2 expression. We thus believe to shed new light into the identification of a potential responder cohort. Importantly, we show that the healthy bone marrow donor population displays comparatively low CB1/2 expression as well. This is important to assess and evaluate the necessary doses and potential side effects.

Due to the excellent safety profile of dronabinol (compare drug information of Marinol®) effective doses are achievable in vivo. However, individual tolerable doses may vary widely—and starting with a sub-effective dose to be increased gradually may be necessary to build up tolerance to the well known psychoactive effects.

In this context, we had the opportunity to extract plasma from an elderly patient treated with dronabinol under palliative supportive care considerations for tumor kachexia. Dronabinol, provided by the university hospital’s pharmacy as 2.5 % oily solution, was started with 2 drops bid and tampered to 6 drops bid without any side effects. The patient was not treated with any antitumor or cytoreductive therapy. Plasma was used to culture Jurkat cells—and a considerable plasma inhibitory effect was documented in an apoptosis assay (Additional file 10: Figure S10). This observation argues for an antileukemic activity of dronabinol in vivo.

Due to sparse densities of cannabinoid receptors in lower brainstem areas, which control cardiovascular and respiratory functions, severe intoxications with THC have rarely been reported [21]. LC_50s_ are not well defined (lethal concentration for male rats were 1270 mg/kg when orally administered; compare http://toxnet.nlm.nih.gov) and dose-limiting side effects may be due to cardiovascular effects by lowering blood pressure and heart rate [22]. In this context it is also important to mention that healthy tissues tend to exhibit lower densities of cannabinoid receptors compared to malignant tissues (see expression data described herein or e.g. Kerner et al. who report on significantly higher CB2 expression in glioblastoma in comparison to healthy brain tissue). These findings suggest that therapeutically relevant and at the same time well tolerated proapoptotic doses can be achieved in acute leukemias.

Importantly, our data is in line with findings of others that have reported on the proapoptotic effect of cannabinoids in leukemia cell lines [22, 23]. Discrepancies for IC_50s_ of THC derivatives reported within different studies are likely due to the known instability and origin of the compound, differences in the chosen time intervals between treatment and analyses, and differing cell culture conditions, including FBS concentrations. In this context, we have previously shown that FBS conditions may have significant impact on in vitro sensitivity profiles of tumor cells towards chemo- or targeted therapeutics, linked to direct drug-protein interactions and indirectly via effects on cell cycle regulation [17, 24]. Thus, our data provides a proof-of-principle, but effective clinical doses will need to be determined in vivo.

Cannabinoid receptor agonists as low-toxic agents may be especially of interest in the context of heavily pre-treated, elderly or therapy refractory disease. Notably, we have evidence that dronabinol retained antileukemic activity in a sample of an otherwise chemotherapy and steroid-refractory ALL patient (see Additional file 11: Figure S11).

In this context, a case report of a 14 year old girl with refractory *BCR-ABL1* (Ph+) ALL was recently published demonstrating dramatic blast reduction in an individual therapy approach using escalating doses of a cannabis extract [25]. It is remarkable, that the selected case fits into the defined responder cohort of our study.

The compiled data demonstrates impressively, that dronabinol should be considered in selected cases of patients with acute leukemia but also stresses on the importance of thoroughly reflecting on the individual expression profiles of CB1/CB2 as well as on additional diagnostic criteria—as e.g. lymphatic markers.

Even though it is not the intended purpose of this article, it should not stay unmentioned that besides the direct anti-leukemic effects of dronabinol the therapeutical use of THC in this patient cohort might exhibit a multitude of positive, desirable side effects like general physical well-being, cachexia control as well as pain, anxiety and stress relief, and thus should facilitate the decision process.

## Conclusion

To summarize, we provide a promising rationale for the clinical use of cannabinoids, such as dronabinol, in distinct entities of acute leukemia—and this approach should further be evaluated.

## Methods

### Cell lines

The CML blast crisis cell line K562, the MLL-AF9 fusion positive acute myelogenous leukemia cell lines MOLM13 and the sister cell line MOLM14, both deriving from the same patient [26], and the human hematopoietic growth factor–dependent M-07e cell line were kindly provided by Drs. Heinrich and Lopez, Oregon Health and Science University, Portland, OR. The acute T-cell lymphoblastic leukemia cell line Jurkat, the AML cell lines HL60 and MV4-11 and the core binding factor leukemia cell line Kasumi1 [27] were obtained from the German Collection of Microorganisms and Cell Cultures (DSMZ).

Cells were cultured in RPMI 1640, supplemented with 10 % fetal bovine serum, 1 % penicillin G (10,000 units/mL), and streptomycin (10,000 μg/mg) (GIBCO/Invitrogen, Darmstadt, Germany or BiochromAG, Berlin, Germany). Negativity for mycoplasma contamination was confirmed using the pluripotent PCR Mycoplasma test kit (AppliChem, Darmstadt, Germany). Cell lines harboring a mutant KIT (Kasumi1), FLT3 (MOLM13; MOLM14, MV4-11) or ABL (K562) isoform were sequence confirmed. M-07e cells were cultured using 10 ng/ml recombinant human granulocyte-macrophage colony stimulating factor (GM-CSF) as a growth supplement.

### Reagents

Dronabinol (i.e. (−)-Δ^9^-Tetrahydrocannabinol, THC), dissolved in methanol, was obtained from THC Pharm (Frankfurt/Main, Germany) with permission of the Federal Opium Agency at the Federal Institute for Drugs and Medical Device, Germany. The selective CB1 antagonist LY320135 and the selective CB2 inverse agonist JTE-907 (CB2) were purchased from Sigma (St. Louis, MO).

### Isolation of bone marrow and peripheral blood mononuclear cells

Bone marrow aspirate and peripheral blood samples from patients with diagnosed acute leukemia were collected in 5000 U heparin after written informed consent, including publication of the data, and approval of the ethics committee of the University of Tübingen. Mononuclear cells were isolated by Ficoll Hypaque density gradient fractionation [17].

### Immunoblotting

Cell pellets were lysed with 100 to 150 μL of protein lysis buffer (50 mmol/L Tris, 150 mmol/L NaCl, 1 % NP40, 0.25 % deoxycholate with added inhibitors aprotinin, AEBSF, leupeptin, pepstatin, sodium orthovanadate, and sodium pyruvate, respectively phosphatase inhibitor cocktails „2“and „1“or „3“(Sigma, St. Louis, MO). Protein from cell lysates (75 to 200 μg protein) was used for whole cell protein analysis after denaturing by Western immunoblot assays using a BioRad Criterion system (protein separation by SDS-PAGE in 3–8 % or 10 % polyacrylamide gels followed by electroblotting onto nitrocellulose membranes). Nonspecific binding was blocked by incubating the blots in nonfat dry milk or BSA. Primary antibodies were incubated for one hour or over night, followed by several washes of Tris-buffered saline (TBS) containing 0.005 % Tween 20. Goat anti-human cannabinoid receptor 1 or 2 (CB1/CB2) antibodies were purchased from Sigma (St. Louis, MO); rabbit anti-human cleaved caspase 3 as well as 9 and rabbit anti-mouse tubulin antibodies were obtained from Cell Signaling Technology (Danvers, MA). The major isoform of CB1 (1a long) has a molecular weight of 52 KDa. The molecular weight of CB2 is 39 KDa – and the corresponding band in the immunoblot for the used antibody is expected at 40-50 KDa according to the manufacturer’s protocol. Donkey anti-goat/rabbit/mouse infrared dye-conjugated secondary antibodies for the LI-COR® imaging detection system were used according to standard protocols (LI-COR Biosciences, Lincoln, NE). Secondary antibodies were applicated for 30‘, followed by several washes. Antibody-reactive proteins were detected using a LI-COR Odyssey® fluorescence optical system (LI-COR Biosciences, Lincoln, NE) [17].

### Apoptosis assays

Translocation of phosphatidylserine from the inner to the outer leaflet of the plasma membrane as an early indicator of apoptosis was analyzed using an annexin V-based assay (Immunotech, Marseilles, France) and a FACScalibur® flow cytometer loaded with CellQuest® analysis software (BD, Heidelberg, Germany) [28].

### Proliferation assays

Cellular proliferation capacity was measured using an 2,3-bis[2-methoxy-4-nitro-5-sulfophenyl]-2H-tetrazolium-5-carboxanilide inner salt (XTT)–based assay (Sigma, MO) [28].

### Immunophenotyping

A routine panel for newly diagnosed acute leukemia was performed for every patient following standard in-house protocols. In addition, rabbit anti-human CB1 or CB2 antibodies (Cell Signaling Technology, Danvers, MA) were conjugated with fluorescent polyclonal secondary anti-rabbit IgG-H&L (FITC) antibodies (Cell Signaling Technology as well) according to the manufacturer protocol and protein expression levels were assessed by flow cytometry using standard protocols.

### Data analysis

Dose-effect plots were created to calculate IC_50s_ using Prism 5.0 Software available from Graph Pad, La Jolla, CA.

## Additional files

Additional file 1: Figure S1. Induction of apoptosis determined by externalization of phosphatidylserine. Jurkat cells are treated with THC for 10 h and analyzed using a flow cytometry annexinV staining protocol. Histograms of representative experiments are provided. (TIFF 432 kb) Additional file 2: Figure S2. Proapoptotic effect of THC is mediated via the mitochondrial intrinsic pathway. Western immunoblotting of cleaved caspase 9 in Jurkat cells treated with THC is shown. Tubulin serves as a loading control. (TIFF 107 kb) Additional file 3: Figure S3. Flow cytometric apoptosis assay. Dose-effect curves for leukemia cell lines treated with THC in a dose-dependent manner are shown. Student’s *t*-test analysis demonstrates significance (p < 0.05) of induction of apoptosis. Experiments were performed in triplicates. Methanol as drug carrier was applicated at the highest tested dose (left panels). Non-linear regression analysis was performed to compute IC_50s_ (right panels). (TIFF 557 kb) Additional file 4: Figure S4. Flow cytometric apoptosis assay. Dose-effect curves for leukemia cell lines treated with THC in a dose-dependent manner are shown. Student’s *t*-test analysis demonstrates significance (p < 0.05) of induction of apoptosis. Experiments were performed in triplicates. Methanol as drug carrier was applicated at the highest tested dose (left panels). Non-linear regression analysis was performed to compute IC_50s_ (right panels). (TIFF 579 kb) Additional file 5: Figure S5. Flow cytometric apoptosis assay. Dose-effect curves for leukemia cell lines treated with THC in a dose-dependent manner are shown. Student’s *t*-test analysis demonstrates significance (p < 0.05) of induction of apoptosis. Experiments were performed in triplicates. Methanol as drug carrier was applicated at the highest tested dose (left panels). Non-linear regression analysis was performed to compute IC50s (right panels). (TIFF 567 kb) Additional file 6: Figure S6. Flow cytometric apoptosis assay. Dose-effect curves for leukemia cell lines treated with THC in a dose-dependent manner are shown. Student’s *t*-test analysis demonstrates significance (p < 0.05) of induction of apoptosis. Experiments were performed in triplicates. Methanol as drug carrier was applicated at the highest tested dose (left panels). Non-linear regression analysis was performed to compute IC50s (right panels). (TIFF 536 kb) Additional file 7: Figure S7. Flow cytometric apoptosis assay. Dose-effect curves for leukemia cell lines treated with THC in a dose-dependent manner are shown. Student’s *t*-test analysis demonstrates significance (p < 0.05) of induction of apoptosis. Experiments were performed in triplicates. Methanol as drug carrier was applicated at the highest tested dose (left panels). Non-linear regression analysis was performed to compute IC50s (right panels). (TIFF 527 kb) Additional file 8: Figure S8. Flow cytometric apoptosis assay. Dose-effect curves for leukemia cell lines treated with THC in a dose-dependent manner are shown. Student’s *t*-test analysis demonstrates significance (p < 0.05) of induction of apoptosis. Experiments were performed in triplicates. Methanol as drug carrier was applicated at the highest tested dose (left panels). Non-linear regression analysis was performed to compute IC50s (right panels). (TIFF 563 kb) Additional file 9: Figure S9. Plasma inhibitory efficacy. Plasma derived from a patient supportively treated with dronabinol (6° bid of a 2.5 % oily solution) for tumor kachexia in a palliative setting was extracted and used to culture Jurkat leukemia cells for 48 and 72 h. Plasma inhibitory efficacy was analyzed in an annexin V/PI-based apoptosis assay. (TIFF 1292 kb) Additional file 10: Figure S10. Reduction of the viable leukemia population upon treatment with THC. Immunphenotyping of the leukemic clone in a FSC/SSC scatter plot was performed in a patient with refractory ALL and >90 % blasts in the peripheral blood. Reduction of the population was followed after exposure to THC for 48 h. Proportion of the remaining viable cell proportion is shown in a dose-effect plot. (TIFF 282 kb) Additional file 11: Figure S11. Sensitivity of Jurkat leukemia cells towards THC after selective CB1-, resp. CB2, CRISPR knockdown. (A) Cells are transfected using standard protocols of the manufacturer (Santa Cruz) using a selective CB1, respectively CB2, CRISPR Double Nickase plasmid. GFP transfection efficiency control by flow cytometry after puromycin selection is shown. EV, empty vector negative control. (B) Validation of CRISPR knockdown of CB1, resp. CB2 protein expression using a flow cytometry approach. (C) Sensitivity of Jurkat cells towards THC (40 μM) after selective CB1, resp. CB2, interference (CB1i/CB2i) with regard to induction of apoptosis. Mean data of 3-5 independent annexin V/PI-based experiments are provided. (*-**) statistical significance at *p* < 0.05 (Student’s *t*-test). EV, empty vector. (TIFF 237 kb)

## Abbreviations

ABL1: Abelson murine leukemia viral oncogene homolog 1
AML: acute myeloid leukemia
ALL: acute lymphoid leukemia
BSA: bovine serum albumin
CB1: cannabinoid receptor 1
CB2: cannabinoid receptor 2
CBFL: core binding factor leukemia
CML: chronic myeloid leukemia
DSMZ: Leibniz Institute, German Collection of Microorganisms and Cell Cultures
FACS: fluorescence-activated cell sorting
FITC: fluorescein isothiocyanate
FLT3: FMS-like tyrosine kinase 3
FSC: forward scatter (distiguishes volume of cells)
IC_50_: concentration sufficient to achieve a 50 % inhibition
IL3: interleukin 3
ITD: internal tandem duplication
KIT: v-kit Hardy-Zuckerman 4 feline sarcoma viral oncogene homolog
LC_50_: lethal concentration killing 50 % ot the cohort
SSC: side scatter (distinguishes granularity of cells and size/shape of nucleus)
PE: R-Phycoerythrin
THC: Delta9-Tetrahydrocannabinol
XTT: 2,3-Bis-(2-methoxy-4-nitro-5-sulfophenyl)-2H-tetrazolium-5-carboxanilid-sodium salt
