# Supplementary figures and images for: Dronabinol has preferential antileukemic activity in acute lymphoblastic and myeloid leukemia with lymphoid differentiation patterns
Source: BMC Cancer. 2016 Jan 16;16:25. doi: 10.1186/s12885-015-2029-8 (PMC4715874; doi:10.1186/s12885-015-2029-8)

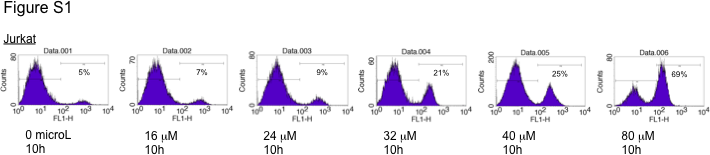

Supplement: Additional file 1: Figure S1. — Induction of apoptosis determined by externalization of phosphatidylserine. Jurkat cells are treated with THC for 10 h and analyzed using a flow cytometry annexinV staining protocol. Histograms of representative experiments are provided. (TIFF 432 kb) [file 12885_2015_2029_MOESM1_ESM.tiff]

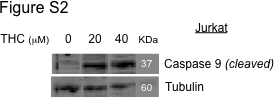

Supplement: Additional file 2: Figure S2. — Proapoptotic effect of THC is mediated via the mitochondrial intrinsic pathway. Western immunoblotting of cleaved caspase 9 in Jurkat cells treated with THC is shown. Tubulin serves as a loading control. (TIFF 107 kb) [file 12885_2015_2029_MOESM2_ESM.tiff]

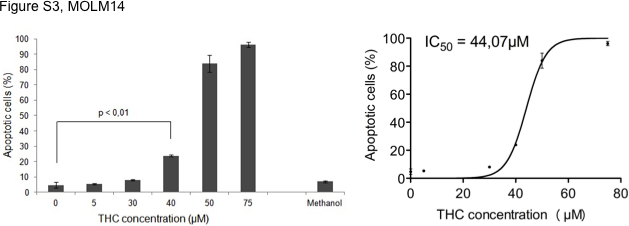

Supplement: Additional file 3: Figure S3. — Flow cytometric apoptosis assay. Dose-effect curves for leukemia cell lines treated with THC in a dose-dependent manner are shown. Student’s t-test analysis demonstrates significance (p < 0.05) of induction of apoptosis. Experiments were performed in triplicates. Methanol as drug carrier was applicated at the highest tested dose (left panels). Non-linear regression analysis was performed to compute IC50s (right panels). (TIFF 557 kb) [file 12885_2015_2029_MOESM3_ESM.tiff]

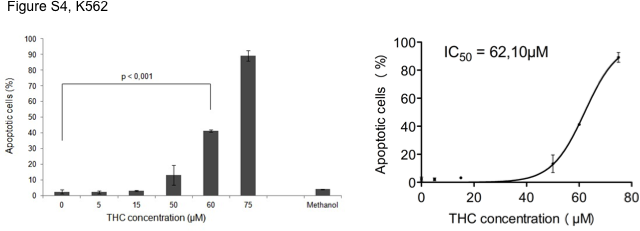

Supplement: Additional file 4: Figure S4. — Flow cytometric apoptosis assay. Dose-effect curves for leukemia cell lines treated with THC in a dose-dependent manner are shown. Student’s t-test analysis demonstrates significance (p < 0.05) of induction of apoptosis. Experiments were performed in triplicates. Methanol as drug carrier was applicated at the highest tested dose (left panels). Non-linear regression analysis was performed to compute IC50s (right panels). (TIFF 579 kb) [file 12885_2015_2029_MOESM4_ESM.tiff]

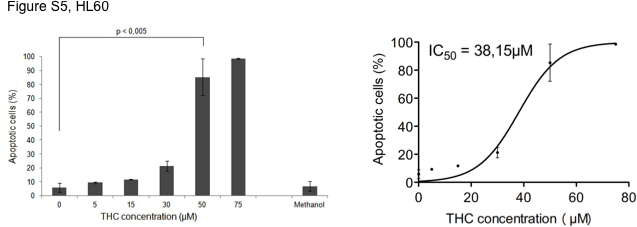

Supplement: Additional file 5: Figure S5. — Flow cytometric apoptosis assay. Dose-effect curves for leukemia cell lines treated with THC in a dose-dependent manner are shown. Student’s t-test analysis demonstrates significance (p < 0.05) of induction of apoptosis. Experiments were performed in triplicates. Methanol as drug carrier was applicated at the highest tested dose (left panels). Non-linear regression analysis was performed to compute IC50s (right panels). (TIFF 567 kb) [file 12885_2015_2029_MOESM5_ESM.tiff]

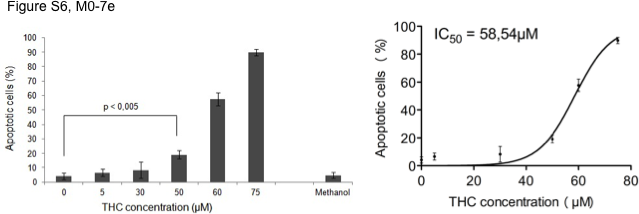

Supplement: Additional file 6: Figure S6. — Flow cytometric apoptosis assay. Dose-effect curves for leukemia cell lines treated with THC in a dose-dependent manner are shown. Student’s t-test analysis demonstrates significance (p < 0.05) of induction of apoptosis. Experiments were performed in triplicates. Methanol as drug carrier was applicated at the highest tested dose (left panels). Non-linear regression analysis was performed to compute IC50s (right panels). (TIFF 536 kb) [file 12885_2015_2029_MOESM6_ESM.tiff]

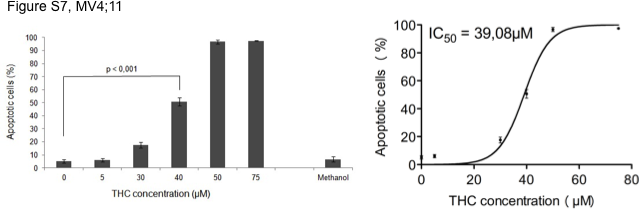

Supplement: Additional file 7: Figure S7. — Flow cytometric apoptosis assay. Dose-effect curves for leukemia cell lines treated with THC in a dose-dependent manner are shown. Student’s t-test analysis demonstrates significance (p < 0.05) of induction of apoptosis. Experiments were performed in triplicates. Methanol as drug carrier was applicated at the highest tested dose (left panels). Non-linear regression analysis was performed to compute IC50s (right panels). (TIFF 527 kb) [file 12885_2015_2029_MOESM7_ESM.tiff]

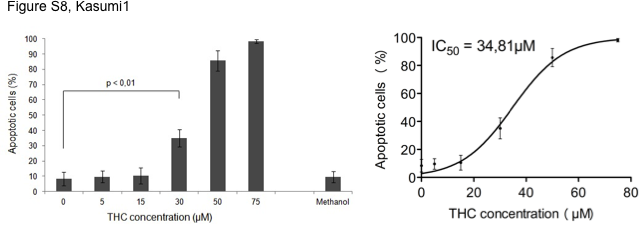

Supplement: Additional file 8: Figure S8. — Flow cytometric apoptosis assay. Dose-effect curves for leukemia cell lines treated with THC in a dose-dependent manner are shown. Student’s t-test analysis demonstrates significance (p < 0.05) of induction of apoptosis. Experiments were performed in triplicates. Methanol as drug carrier was applicated at the highest tested dose (left panels). Non-linear regression analysis was performed to compute IC50s (right panels). (TIFF 563 kb) [file 12885_2015_2029_MOESM8_ESM.tiff]

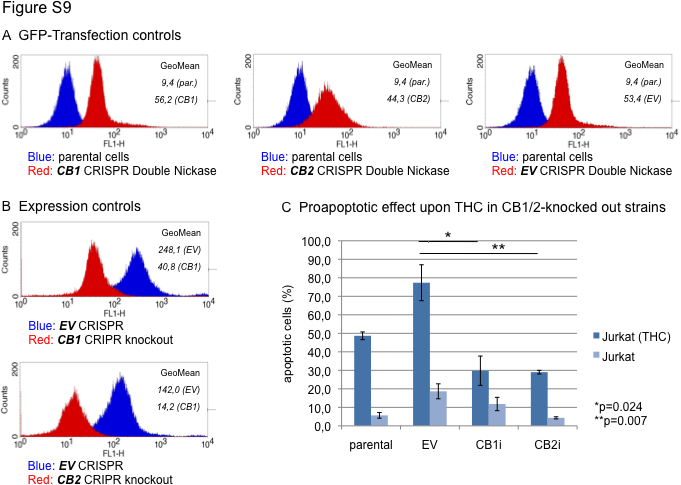

Supplement: Additional file 9: Figure S9. — Plasma inhibitory efficacy. Plasma derived from a patient supportively treated with dronabinol (6° bid of a 2.5 % oily solution) for tumor kachexia in a palliative setting was extracted and used to culture Jurkat leukemia cells for 48 and 72 h. Plasma inhibitory efficacy was analyzed in an annexin V/PI-based apoptosis assay. (TIFF 1292 kb) [file 12885_2015_2029_MOESM9_ESM.tiff]

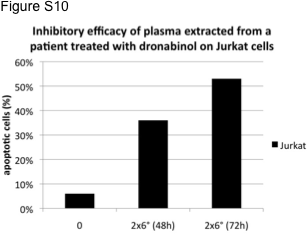

Supplement: Additional file 10: Figure S10. — Reduction of the viable leukemia population upon treatment with THC. Immunphenotyping of the leukemic clone in a FSC/SSC scatter plot was performed in a patient with refractory ALL and >90 % blasts in the peripheral blood. Reduction of the population was followed after exposure to THC for 48 h. Proportion of the remaining viable cell proportion is shown in a dose-effect plot. (TIFF 282 kb) [file 12885_2015_2029_MOESM10_ESM.tiff]

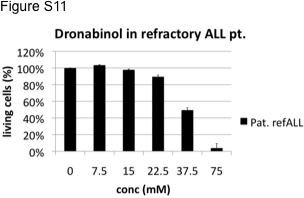

Supplement: Additional file 11: Figure S11. — Sensitivity of Jurkat leukemia cells towards THC after selective CB1-, resp. CB2, CRISPR knockdown. (A) Cells are transfected using standard protocols of the manufacturer (Santa Cruz) using a selective CB1, respectively CB2, CRISPR Double Nickase plasmid. GFP transfection efficiency control by flow cytometry after puromycin selection is shown. EV, empty vector negative control. (B) Validation of CRISPR knockdown of CB1, resp. CB2 protein expression using a flow cytometry approach. (C) Sensitivity of Jurkat cells towards THC (40 μM) after selective CB1, resp. CB2, interference (CB1i/CB2i) with regard to induction of apoptosis. Mean data of 3-5 independent annexin V/PI-based experiments are provided. (*-**) statistical significance at p < 0.05 (Student’s t-test). EV, empty vector. (TIFF 237 kb) [file 12885_2015_2029_MOESM11_ESM.tiff]
